# The association between dietary fatty acid intake and the risk of developing preeclampsia: a matched case–control study

**DOI:** 10.1038/s41598-021-83674-3

**Published:** 2021-02-18

**Authors:** Shu-na Li, Yan-hua Liu, Ze-yan Luo, Yun-feng Cui, Yuan Cao, Wen-jun Fu, Wei-feng Dou, Dan-dan Duan, Xian-lan Zhao, Yu-ming Chen, Quan-jun Lyu, Qing-shan Chen, Fang-fang Zeng

**Affiliations:** 1 Department of Epidemiology, School of Medicine, Jinan University, No.601 Huangpu Road West, Guangzhou, 510632 Guangdong China; 2 Department of Nutrition, the First Affiliated Hospital of Zhengzhou University, No. 1 Jianshe East Road, Zhengzhou, 450052 China; 3 Department of Nutrition and Food Hygiene, College of Public Health, Zhengzhou University, Zhengzhou, 450001 China; 4 Department of Obstetrics, the First Affiliated Hospital of Zhengzhou University, Zhengzhou, 450052 China; 5 Department of Clinical Nutrition, New Area People’s Hospital of Luoyang, Luoyang, 471023 China; 6 Guangdong Provincial Key Laboratory of Food, Nutrition and Health, School of Public Health, Sun Yat-Sen University, Guangzhou, 510080 China

**Keywords:** Diseases, Risk factors

## Abstract

The association between dietary fat intake during pregnancy and the risk of developing preeclampsia has been examined in many epidemiological studies, but the results remain inconsistent. The aim of this study was to clarify this association in pregnant Chinese women. After conducting 1:1 matching, 440 pairs consisting of pregnant women with preeclampsia and hospital-based, healthy pregnant women matched by gestational week (± 1 week) and age (± 3 years) were recruited. A 79-item semi-quantitative food frequency questionnaire administered during face-to-face interviews was used to estimate the participants’ dietary intake of fatty acids. We found that the intakes of arachidonic acid (AA), eicosapentaenoic acid (EPA), and docosahexaenoic acid (DHA) were inversely associated with the risk of developing preeclampsia. Compared with the lowest quartile intake, the multivariate-adjusted odds ratios (95% confidence interval) of the highest quartile intake were 0.42 (0.26–0.68, *p-*trend < 0.001) for EPA, 0.52 (0.3–0.83, *p-*trend = 0.005) for DHA, and 0.41 (0.19–0.88, *p-*trend = 0.007) for AA. However, we did not observe any significant associations between the intake of total fatty acids, saturated fatty acids, and mono-unsaturated fatty acids and the risk of developing preeclampsia. Our results showed that the dietary intake of long-chain polyunsaturated fatty acids (i.e., EPA, DHA, and AA) may protect pregnant Chinese women against the development of preeclampsia.

## Introduction

Preeclampsia is a multi-system disorder particular to pregnancy, and is characterized by the onset of hypertension and proteinuria after the twentieth week of gestation^1^. In the absence of proteinuria, preeclampsia is often diagnosed as hypertension associated with thrombocytopenia, impaired liver function, renal insufficiency, pulmonary edema or new-onset cerebral or visceral disturbances^2^. Preeclampsia is a key cause of maternal and perinatal death and morbidity worldwide^3^. The World Health Organization Multicountry Survey on maternal and newborn health reported an overall global incidence of preeclampsia of 2.61%^4^. Preeclampsia accounts for 3–4% of all adverse effects of pregnancy, and women who experienced preeclampsia in their first pregnancy or two most recent pregnancies face a 14.7% and 31.9% risk, respectively, of developing preeclampsia in subsequent pregnancies^5^. Therefore, determining the risk factors for the development of preeclampsia and outlining measures to reduce its incidence are important for ensuring the health of mothers and their babies.

Fatty acids are an important part of the diet and may play a significant role in the development of preeclampsia. The dietary intake of saturated fatty acids (SFAs), especially lauric, myristic, and palmitic acids, may increase concentrations of total cholesterol and low-density lipoprotein (LDL) cholesterol^6–8^. SFAs are also known to increase coagulation and promote inflammation^8^. Monounsaturated fatty acids (MUFAs), such as oleic acid, can slightly lower blood pressure and improve glucose control and insulin sensitivity^9,10^. Long-chain polyunsaturated fatty acids (PUFAs), including n-3 and n-6 PUFAs, play an important role in many aspects of human health, such as maintaining the structure and function of organs, and neurological, psychological, and psychiatric health.

A recently conducted meta-analysis showed that dietary supplementation with n-3 fatty acids can protect women with low-risk pregnancies against the risk of developing preeclampsia^11^. Concentrations of major n-6 and n-3 PUFAs in erythrocytes and umbilical vessels were found to be lower in women with preeclampsia than in healthy controls^12,13^. However, other studies do not support these observations^14^.

Observational epidemiological studies have also assessed the link between dietary fatty acids, which reflect actual intake, and the risk of developing preeclampsia, but the findings were inconsistent. A prospective cohort study in Denmark that included 65,522 pregnancies and 1,302 cases of preeclampsia revealed that a higher intake of a long-chain omega-3 fatty acid (docosahexaenoic acid [DHA]) was negatively associated with preeclampsia^15^. Other studies with limited sample sizes (generally fewer than 100 participants) found a positive link between high SFA intake and preeclampsia: SFA and MUFA concentrations were higher in the maternal plasma of women with preeclampsia, whereas PUFA concentrations were lower^16,17^. However, these associations have not been established in pregnant Asian women, due to their dietary patterns being different from those of pregnant Western women. A global survey of healthy adults reported that blood concentrations of n-3 long-chain PUFAs (specifically eicosapentaenoic acid [EPA] and DHA) differed across countries and regions. For example, Asians (Chinese, Russians, and Singaporeans) had lower blood concentrations of EPA and DHA, whereas Swedes, Tunisians, and Chileans had moderate blood concentrations of EPA and DHA^18^.

To further explore the relationship between dietary fatty acids and preeclampsia in pregnant Chinese women, we carried out a 1:1 matched case–control study that included 440 pregnant participants diagnosed with preeclampsia and 440 normal pregnant women.

## Result

### Baseline characteristics of included participants

The demographics and lifestyle characteristics of cases and controls are presented in Table 1. There were no significant differences between preeclampsia cases and controls in terms of age (cases vs. controls: 30.88 ± 5.03 years vs. 31.03 ± 4.85 years) and gestational week (cases vs. controls: 34.17 ± 2.90 weeks vs. 34.24 ± 2.67 weeks). The number of participants who had higher education (college/university or above) and a BMI < 24 kg/m^2^ were higher in the control group than in the case group (*p* < 0.05). The total energy intake was higher in controls (1962.08 ± 520.64 kcal/d) than in cases (1850.39 ± 504.27 kcal/d).

**Table 1:** Demographics, lifestyle characteristics, and select preeclampsia risk factors among pregnancy women.

Variables	Case (n = 440)	Control (n = 440)	*P* value
Age (years), mean (SD)	30.88 (5.03)	31.03 (4.85)	0.114
Gestational (weeks), mean (SD)	34.17 (2.90)	34.24 (2.67)	0.066
**Education level, N (%)**			0.005
Primary school or less	44 (10.0)	18 (4.1)	
Secondary / high school	238 (54.1)	229 (52.2)	
College / university or above	158 (35.9)	192 (43.7)	
**Household income (yuan/per month/ person), N (%)**			0.405
< 2000	61 (14.7)	46 (11.0)	
2000 to 4000	216 (52.2)	211 (50.2)	
4000 to 6000	78 (18.8)	82 (19.5)	
> 6000	59 (14.3)	81 (19.3)	
**Pre-pregnancy BMI (kg/m**^**2**^)*, N (%)			< 0.001
< 24	272 (61.8)	341 (77.5)	
24 to 27.9	102 (23.2)	66 (15.0)	
≥ 28	66 (15.0)	33 (7.5)	
**Alcohol drinking, N (%)**			1.000
No	430 (97.7)	431 (98.0)	
Yes	10 (2.3)	9 (2.0)	
**Passive smoking†, N (%)**			0.499
No	208 (47.6)	220 (50.1)	
Yes	229 (52.4)	219 (49.9)	
Total energy intake (kcal/d), mean (SD)	1850.39 (504.27)	1962.08 (520.64)	0.001
Physical activity (MET/ h·d)‡, mean (SD)	26.94 (3.95)	26.60 (4.47)	0.241
**Multivitamin use, N (%)**			0.497
No	384 (87.3)	376 (85.5)	
Yes	56 (12.7)	64 (14.5)	
**Folic acid supplement, N (%)**			0.543
No	89 (20.2)	81 (18.4)	
Yes	351 (79.8)	359 (81.6)	

Abbreviation: SD, standard deviation; BMI: body mass index.

*: BMI of gestational age of 28 weeks or more.

^†^: Passive smoking refers to the husband smokes near the pregnant woman in the first three months of pregnancy.

^‡^: Physical activities included daily occupational, leisure-time and household-chores, evaluated by metabolic equivalent (MET) hours per day.

Continuous variables were described by mean (SD), evaluated by t-tests; Categorical variables were described by number (%), evaluated by chi-square tests.

### Diet intake in case and control groups

The daily intake of dietary nutrients in cases and controls after adjusting for energy are shown in Table 2. The median intake (interquartile range [IQR]) of fatty acid was 59.8 g/d (51.81–68.14) in the preeclampsia group and 60.73 g/d (51.92–68.91) in the control group, and there was no significant difference in the median intake between groups (*p* = 0.769). In addition, there were no differences between case and control groups in terms of dietary intake of SFAs, MUFAs, PUFAs, n-6 PUFAs, and n-3 PUFAs (all *p* > 0.05). However, the intake of arachidonic acid (AA), EPA, DHA, and total cholesterol was significantly lower in the case group than in the control group (all *p* < 0.001).

**Table 2:** Daily intake of dietary nutrients after energy-adjusted among preeclampsia cases and controls.

	Case (n = 440)		Control (n = 440)		*P* value
	Median	IQR	Median	IQR	
**Total fatty acid (g/d)**	59.80	51.81–68.14	60.73	51.92–68.91	0.769
SFA (g/d)	16.55	14.14–19.13	16.92	14.39–19.48	0.221
MUFA (g/d)	25.05	20.03–29.06	24.63	20.10–28.92	0.951
PUFA (g/d)	21.14	16.76–25.72	20.32	16.77–25.27	0.306
n-6 PUFA	15.20	11.82–18.57	14.91	11.55 -`18.28	0.731
LA (g/d)	15.13	11.77–18.52	14.81	11.44–18.21	0.703
AA (mg/d)	63.20	36.82–87.50	72.92	50.45–101.85	< 0.001
n-3 PUFA	1.55	0.75–2.40	1.59	0.82–2.31	0.671
ALA (g/d)	1.54	0.72–2.38	1.58	0.79–2.31	0.682
EPA (mg/d)	2.45	0.70–6.40	4.70	1.33–10.60	< 0.001
DHA (mg/d)	2.30	0.70–6.70	3.50	1.30–8.60	< 0.001
Total cholesterol (mg/d)	361.48	202.40–497.32	407.88	281.73–571.58	< 0.001
Carbohydrate (g/d)	248.51	227.98–268.83	242.67	221.66–264.50	0.083

Abbreviation: IQR: interquartile range; SFA, saturated fatty acids; MUFA, mono-unsaturated fatty acids; PUFA, polyunsaturated fatty acids; LA, linoleic acid; AA, arachidonic acid; ALA, alpha-linolenic acid; EPA, eicosapentaenoic acid; DHA, docosahexaenoic acid.

Median values and 25^th^, 75^th^ percentiles.

### Association between fatty acids and the risk of developing preeclampsia

As shown in Table 3, no significant associations were found between the intake of total fatty acids, SFAs, PUFAs, ratio of MUFAs to SFAs, or n-3 PUFAs and the risk of developing preeclampsia, both with and without adjustment of the covariates (all *p-*trends > 0.05).

**Table 3:** Risk of preeclampsia during pregnancy according to quartiles of dietary fatty acids intake.

Fat	Quartiles of dietary energy-adjusted intake				
	Q1	Q2	Q3	Q4	*P*-trend
**Total fatty acid**					
n (case/control)	110/110	116/104	108/112	106/114	
Median, g/d (case/control)	43.70/44.97	57.15/56.20	63.97/64.41	75.10/74.26	
Crude OR (95% CI)	1.00	1.11 (0.76–1.62)	0.97 (0.67–1.40)	0.94 (0.64–1.37)	0.554
Adjusted OR (95% CI)	1.00	1.37 (0.83–2.27)	1.32 (0.75–2.30)	1.40 (0.69–2.83)	0.485
**SFA**					
n (case/control)	114/106	115/105	107/113	104/116	
Median, g/d (case/control)	12.34/12.48	15.54/15.59	17.84/18.10	21.05/21.51	
Crude OR (95% CI)	1.00	1.01 (0.69–1.48)	0.89 (0.60–1.30)	0.85 (0.58–1.23)	0.267
Adjusted OR (95% CI)	1.00	1.19 (0.74–1.90)	1.35 (0.81–2.24)	1.65 (0.92–2.97)	0.089
**MUFA**					
n (case/control)	111/109	100/120	118/102	111/109	
Median, g/d (case/control)	15.80/17.35	22.55/22.92	26.50/26.87	32.68/32.76	
Crude OR (95% CI)	1.00	0.81 (0.56–1.18)	1.16 (0.79–1.70)	1.00 (0.69–1.46)	0.582
Adjusted OR (95% CI)	1.00	1.00 (0.61–1.64)	1.50 (0.87–2.58)	1.52 (0.82–2.83)	0.081
**PUFA**					
n (case/control)	110/110	99/121	117/103	114/106	
Median, g/d (case/control)	14.27/14.22	18.67/18.63	22.61/22.76	29.67/28.17	
Crude OR (95% CI)	1.00	0.82 (0.56–1.20)	1.10 (0.76–1.61)	1.09 (0.74–1.61)	0.353
Adjusted OR (95% CI)	1.00	0.66 (0.41–1.05)	1.10 (0.68–1.79)	0.94 (0.55–1.60)	0.537
**Ratio of MUFA to SFA**					
n (case/control)	110/110	102/118	104/116	124/96	
Median (case/control)	1.14/1.14	1.39/1.39	1.57/1.56	1.86/1.84	
Crude OR (95% CI)	1.00	0.87 (0.60–1.26)	0.92 (0.63–1.34)	1.31 (0.89–1.93)	0.170
Adjusted OR (95% CI)	1.00	0.80 (0.52–1.24)	0.76 (0.49–1.18)	1.15 (0.72–1.82)	0.593

Abbreviation: OR, odds ratio; CI, confidence interval; SFA, saturated fatty acids; MUFA, mono-unsaturated fatty acids; PUFA, polyunsaturated fatty acids.

Crude and adjusted OR (95% CI): from conditional logistic model. Covariates includes age, gestational weeks, education level, household income, pre-pregnancy body mass index, alcohol drinking, passive smoking, use of multivitamin, use of folic acid supplement, daily energy intake, carbohydrate intake and total cholesterol intake by enter method.

**Figure 1:**
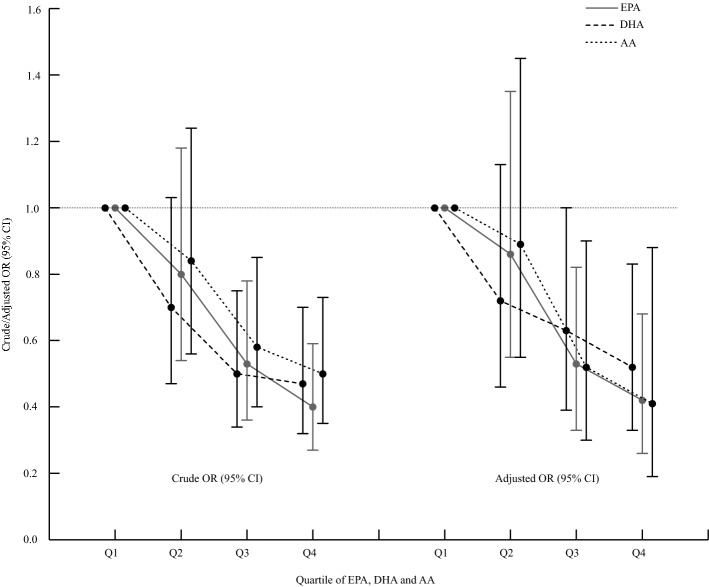
The risk of preeclampsia based on the intake of dietary EPA, DHA, and AA intake levels; Error bars indicate 95% confidence intervals. Abbreviations: EPA, eicosapentaenoic acid; DHA, docosahexaenoic acid; AA, arachidonic acid; 95%CI, 95% confidence intervals.

Table 4 shows our assessment of the relationship between the PUFA components and the risk of developing preeclampsia. The intake of n-3 PUFAs (including alpha-linolenic acid [ALA]), n-6 PUFAs (including linoleic acid [LA]), and n-3/n-6 PUFAs was not associated with the risk of developing preeclampsia. In contrast, significant inverse and dose–response associations were found for dietary AA, EPA, and DHA intake in both univariate and multivariate models. Compared with the lowest quartiles, the adjusted odd ratios (ORs) (95% confidence intervals (CIs)) of preeclampsia for the highest quartile were 0.41 (0.19–0.88, *p-*trend = 0.007) for AA, 0.42 (0.26–0.68, *p-*trend < 0.001) for EPA, and 0.52 (0.33–0.83, *p-*trend = 0.005) for DHA (Table 4, Fig. 1).

**Table 4:** Risk of preeclampsia during pregnancy according to quartiles of different types of polyunsaturated fatty acids.

	Quartiles of dietary energy-adjusted intake				
	Q1	Q2	Q3	Q4	*P*-trend
**n-6 PUFAs**					
n (case/control)	108/112	107/113	111/109	114/106	
Median, g/d (case/control)	9.61/9.84	13.45/13.42	16.23/16.75	21.28/21.74	
Crude OR (95% CI)	1.00	0.98 (0.67–1.42)	1.05 (0.73–1.53)	1.12 (0.77–1.63)	0.506
Adjusted OR (95% CI)	1.00	1.12 (0.71–1.76)	0.95 (0.60–1.50)	1.21 (0.73–2.00)	0.656
**LA**					
n (case/control)	108/112	104/116	114/106	114/106	
Median, g/d (case/control)	9.58/9.76	13.36/13.40	16.16/16.71	21.22/21.64	
Crude OR (95% CI)	1.00	0.93 (0.64–1.35)	1.11 (0.77–1.62)	1.12 (0.77–1.63)	0.397
Adjusted OR (95% CI)	1.00	1.07 (0.68–1.68)	0.99 (0.63–1.58)	1.22 (0.74–2.01)	0.546
**AA**					
n (case/control)	130/90	121/99	99/121	90/130	
Median, mg/d (case/control)	25.88/22.92	57.41/57.37	78.35/77.64	114.81/114.58	
Crude OR (95% CI)	1.00	0.84 (0.56–1.24)	0.58 (0.40–0.85)	0.50 (0.35–0.73)	< 0.001
Adjusted OR (95% CI)	1.00	0.89 (0.55–1.45)	0.52 (0.30–0.90)	0.41 (0.19–0.88)	0.007
**n-3 PUFAs**					
n (case/control)	115/105	109/111	103/117	113/107	
Median, g/d (case/control)	0.52/0.58	1.21/1.23	1.95/1.96	2.92/2.91	
Crude OR (95% CI)	1.00	0.91 (0.63–1.31)	0.81 (0.56–1.17)	0.97 (0.66–1.42)	0.712
Adjusted OR (95% CI)	1.00	0.98 (0.64–1.51)	0.78 (0.50–1.20)	0.87 (0.54–1.38)	0.349
**ALA**					
n (case/control)	115/105	109/111	103/117	113/107	
Median, g/d (case/control)	0.50/0.57	1.19/1.21	1.92/1.95	2.91/2.90	
Crude OR (95% CI)	1.00	0.91 (0.63–1.32)	0.81 (0.56–1.17)	0.97 (0.66–1.42)	0.711
Adjusted OR (95% CI)	1.00	0.97 (0.63–1.49)	0.77 (0.50–1.19)	0.86 (0.54–1.36)	0.328
**EPA**					
n (case/control)	134/86	122/98	99/121	85/135	
Median, mg/d (case/control)	0.24/0.31	2.15/1.94	4.98/5.57	15.11/14.72	
Crude OR (95% CI)	1.00	0.80 (0.54–1.18)	0.53 (0.36–0.78)	0.40 (0.27–0.59)	< 0.001
Adjusted OR (95% CI)	1.00`	0.86 (0.55–1.35)	0.53 (0.33–0.82)	0.42 (0.26–0.68)	< 0.001
**DHA**					
n (case/control)	133/87	116/104	97/123	94/126	
Median, mg/d (case/control)	0.13/0.08	1.87/2.01	4.74/4.36	13.65/13.20	
Crude OR (95% CI)	1.00	0.70 (0.47–1.03)	0.50 (0.34–0.75)	0.47 (0.32–0.70)	< 0.001
Adjusted OR (95% CI)	1.00	0.72 (0.46–1.13)	0.63 (0.39–1.00)	0.52 (0.33–0.83)	0.005
**n-3/n-6 PUFAs**					
n (case/control)	116/104	108/112	101/119	115/105	
Median (case/control)	0.03/0.03	0.09/0.08	0.12/0.13	0.23/0.24	
Crude OR (95% CI)	1.00	0.87 (0.60–1.27)	0.76 (0.52–1.11)	1.00 (0.67–1.48)	0.981
Adjusted OR (95% CI)	1.00	0.94 (0.59–1.50)	0.67 (0.43–1.04)	1.03 (0.65–1.62)	0.650

Abbreviation: PUFAs, polyunsaturated fatty acids; OR, odds ratio; LA, linoleic acid; AA, arachidonic acid; ALA, alpha-linolenic acid; EPA, eicosapentaenoic acid; DHA, docosahexaenoic acid.

Crude and adjusted OR (95% CI): from conditional logistic model. Covariates includes age, gestational weeks, education level, household income, pre-pregnancy body mass index, alcohol drinking, passive smoking, use of multivitamin, use of folic acid supplement, daily energy intake, carbohydrate intake and total cholesterol intake by enter method.

We also performed sensitivity analyses to scrutinize the robustness of our results, by excluding 58 pairs of participants who had gestational diabetes mellitus (GDM). The previously observed associations persisted for the intake of total fatty acids, SFAs, MUFAs, PUFAs, EPA, and DHA, but the effects of AA were no longer visible (adjusted OR for Q4 vs. Q1: 0.85, 95% CI: 0.30–2.45, *p-*trend = 0.529) (Supplementary Materials: Tables S1 and S2).

## Discussion

The results of this 1:1 matched case–control study, which included 440 pairs of women with preeclampsia and healthy controls, showed that the dietary intake of n-3 PUFAs (mainly EPA and DHA) and n-6 PUFAs (mainly AA) was negatively associated with the risk of developing preeclampsia. However, no significant associations were observed between the dietary intake of total fatty acids, SFAs, and MUFAs and the risk of developing preeclampsia. These associations, aside from that with AA intake, persisted after excluding participants with GDM.

Previous studies have assessed the association between different fatty acids and the risk of developing preeclampsia. Nandi et al*.*^19^ observed that maternal plasma PUFA concentrations were lower and those of SFAs and MUFAs are higher in women with preeclampsia (all *p* < 0.05). Cord erythrocyte PUFA concentrations are also higher in women with preeclampsia (*p* < 0.01). Our findings concur with those of some previous epidemiology studies, in that the dietary intake of SFAs, MUFAs, PUFAs, total n-6 PUFAs, and total n-3 PUFAs were not associated with the development of preeclampsia^15^. In contrast, a case–control study of pregnant Jordanian women found that the risk of developing preeclampsia was associated with a high dietary intake of saturated fat^16^. These inconsistencies might arise from differences in demographics, sample size, and food source.

The association between n-3 PUFAs, particularly DHA and EPA, and preeclampsia has been explored by different studies, but the conclusions remain inconsistent. In one study, the plasma concentrations of n-3 PUFAs, EPA, and DHA were found to be lower in subjects with preeclampsia than in control subjects^20^. In addition, the umbilical arteries and veins in women with preeclampsia contain lower amounts of n-3 PUFAs^13^. Moreover, lower concentrations of n-3 PUFAs (including EPA and DHA) in erythrocytes were associated with an increased risk of developing preeclampsia^21^; these findings are also supported by our study. In our study, we found no evidence for a significant association between the dietary intake of total n-3 PUFAs and the risk of developing preeclampsia. However, we did observe a negative association between the dietary intake of DHA, EPA, and the risk of developing preeclampsia (*p*-trend < 0.05). Similarly, a large prospective cohort study conducted by the Danish National Birth Cohort (1996–2012), which included 1,302 women with preeclampsia, demonstrated that women whose DHA intake was in the top quintile had a lower risk of developing preeclampsia (relative risk [RR] = 0.46, 95% CI: 0.25–0.83) and severe preeclampsia (RR = 0.67, 95% CI: 0.51–0.89) than women whose intake was in the bottom quintile. They also reported that EPA could reduce the risk of developing preeclampsia (Q5 vs. Q1: OR = 0.66, 95% CI: 0.34–1.27) and severe preeclampsia (Q5 vs. Q1: OR = 0.74, 95% CI: 0.56–0.99)^15^. Oken et al*.*^22^ found that the risk of developing preeclampsia decreased if the intake of DHA and EPA was increased (OR = 0.84, 95% CI: 0.69–1.03 per 100 mg/day), which is consistent with our findings. A recent meta-analysis conducted by Bakouei et al*.*^11^ illustrated that n-3 PUFA supplementation lowered the risk of developing preeclampsia (RR = 0.82, 95% CI, 0.70–0.97, *p* = 0.024, *I*^2^ = 19.0%). However, they found no evidence for this association in pregnant women whose dietary fatty acid intake was restricted to DHA (RR = 0.84, 95% CI, 0.6–1.18, *p* = 0.308, *I*^2^ = 0%), which is inconsistent with our findings.

Some studies have reached different conclusions on the association of the risk of developing preeclampsia with the dietary intake of specific n-6 PUFAs, such as AA. We found a negative association between the dietary intake of AA and the risk of developing preeclampsia (adjusted OR for Q4 vs. Q1: 0.39, 95% CI: 0.18–0.81, *p-*trend = 0.005), which are inconsistent with the results of previous studies. A case–control study (99 pregnant women with preeclampsia and 100 normotensive pregnant women) conducted in Lima, Peru, was unable to provide clear evidence for an association between the dietary intake of AA and the risk of developing preeclampsia^21^. Other studies have reported that erythrocyte AA concentrations are higher in the third trimester of pregnancy in preeclampsia cases^12,23^. In addition, biochemical studies showed that women with preeclampsia have lower colostrum concentrations of AA than normotensive women^24^. Wadhwani et al*.*^25^ reported that the maternal erythrocyte AA proportions were lower at the time of delivery in preeclampsia cases than in healthy controls. The results of Wang et al*.*^20^ also demonstrate that placental tissues AA concentrations are lower in women with preeclampsia than in normal pregnancies.

Despite the above results, we found that the effects of the dietary intake of AA no longer held after adjusting the covariates in the sensitivity analysis. We found that total cholesterol, which was a correction factor, had a significant effect on the association between the dietary intake of AA and the risk of developing preeclampsia (after removing the correction factor for cholesterol, adjusted *p*-trend < 0.05). Therefore, we speculated that there may be some links between total cholesterol, AA, GDM, and preeclampsia, but the exact mechanism behind this relationship requires further clarification.

Certain subtypes of fatty acids, especially PUFAs (n-3 and n-6 PUFAs), may have specific roles in the mechanisms that underpin the association of PUFA and the risk of developing preeclampsia, although how these molecules exert their effects remain to be determined. It is well known that dietary intake is the major source of PUFAs detected in systemic circulation^20^. Related studies have confirmed that the fatty acid composition of maternal plasma, liver, placenta, and the fetus are highly dependent on the dietary fatty acid intake of the mother^26^. Recent reports suggest that placental inflammation and oxidative stress might play a role in the pathology of preeclampsia^27–29^. It has been reported that n-3 PUFAs have anti-oxidative and anti-inflammatory properties^26,30,31^, which might further decrease the risk of developing preeclampsia. Jones et al*.*^26,32^ found that maternal n-3 PUFAs inhibit oxidative damage by lowering the placental concentrations of F_2_-isoprostanes, which are reliable markers of oxidative damage, and by either enhancing ROS clearance or limiting ROS generation. The resolution of inflammation is mainly driven by resolvins, protectins, maresins, and lipoxins, which are specialized pro-resolving lipid mediators. DHA is the only source of protectins and maresins, whereas resolvins are derived from EPA or DHA. Furthermore, Peng et al.^33^ conducted a related experiment on mice, in which male and female SIRT1^+/−^ were allowed to mate at night, and the primiparous SIRT1^+/−^ mice were then fed either a 60% kcal high-fat diet or an energy-equivalent EPA diet (4.4% EPA-ethyl ester). They found that the EPA diet significantly lowered the plasma concentrations of the inflammatory factors interleukin-6 and tumor necrosis factor α, which can influence placental development and increase the risk of developing preeclampsia. DHA possibly reduces the risk of developing preeclampsia by reducing the expression of vascular cell adhesion protein, and thus decreasing vascular damage^15,34^.

A key feature of preeclampsia is autoimmune dysfunction caused by the generation of autoantibodies (e.g., angiotensin II type 1 receptor autoantibody [AT1-AA]) during pregnancy^35–41^. A recent study on mouse knockout and trophoblast models has shown that lipoxin A_4_ (LXA_4_) can suppress and/or restrict AT1-AA production by modulating the activity of caspase-1^42^. Given that AA is the precursor of LXA_4_, AA might affect the risk of developing preeclampsia by affecting the concentration of LXA_4_.

Some limitations should be acknowledged in our study. First, the inherent disadvantage of case–control studies is that they cannot clearly discern causality. Second, the information we obtained on dietary intake was based on the participants’ recollection of their diet three months prior to the study period, which may have been affected by recall bias that limited the accuracy of our results. To mitigate this effect, we performed face-to-face interviews and presented the participants with photographs of specific food portions and weights to help them to recollect their daily diets. Third, although we adjusted for possible confounding variables to analyze the association between dietary fatty acid intake and the risk of developing preeclampsia, potential unknown factors may influence the results. Fourth, we did not measure the blood and placenta concentrations of key nutrients (such as fatty acids) in our study, which means that we lacked biochemical evidence to support our conclusions. Finally, we did not obtain information on any medication used by the participants for the treatment of preeclampsia, which could further affect the results.

## Conclusion

We found that higher dietary intake of DHA, EPA, and AA were associated with a lower risk of developing preeclampsia during pregnancy. We were unable to find similar evidence for an association of dietary intake of total fatty acids, SFAs, and MUFAs with the risk of developing preeclampsia. Further prospective studies with larger sample sizes are needed to confirm this association in pregnant Chinese women.

## Method

### Study population

This 1:1 matched case–control study was performed in the First Affiliated Hospital of Zhengzhou University, China, between March 2016 and June 2019, to assess the association between dietary fatty acid intake and the risk of developing preeclampsia. The design of the study has been previously described in detail^43^. Briefly, preeclampsia patients were women experiencing a singleton pregnancy, over 18 years of age, and were at least at 28 weeks of gestation. We excluded those with a history of malignancy, hyperthyroidism, heart disease, immune system diseases, chronic renal insufficiency, other endocrine system diseases that may lead to changes in dietary habit, depression, and other mental or cognitive disorders. Cases were defined as women diagnosed with preeclampsia based on China’s ‘Diagnosis and treatment guideline of hypertensive disorders in pregnancy (2015)’ guide^44^. In this guide, a woman is diagnosed with preeclampsia when her systolic blood pressure (SBP) is ≥ 140 mmHg and/or her diastolic blood pressure (DBP) is ≥ 90 mmHg after 20 weeks of gestation. In addition, she should display one of the following characteristics: (1) urinary protein ≥ 0.3 g/24 h, or urinary protein to creatinine ratio ≥ 0.3, or random urinary protein ≥ ( +) (as the examination for quantitative urine protein cannot be carried out in pregnant women); (2) non-albuminuria, accompanied by damage to organs or systems such as the heart, lung, liver, kidney, or other important organs, and abnormal changes in the circulatory system, digestive system, or nervous system, or placental-fetal involvement.

Control subjects were women who were prepared for delivery in the same hospital, who did not have hypertension or proteinuria, and were matched with corresponding cases for age (± 3 years), week of gestation (± 1 week), and GDM. The same exclusion criteria for the preeclampsia cases applied to the control participants.

The sample size was estimated using α = 0.05 and a power of 80% (β = 0.2). We assumed that 25% of the general population had high dietary intake of fatty acid (e.g., DHA), allowing us to estimate the OR between fatty acid (e.g., DHA) intake and the risk of developing preeclampsia risk to be 0.46^15^. Our sample size of 209 cases was calculated based on the above assumptions.

### Ethics declarations

This study was approved by the Ethics Committee of Scientific Research and Clinical Trials of the First Affiliated Hospital of Zhengzhou University (No. Scientific research-2016-LW-34). All the participants signed written informed consent before epidemiological data and biological specimens were collected. All of the procedures were performed in accordance with the Declaration of Helsinki guidelines and regulations.

### Data collection

A structured questionnaire was designed and a face-to-face interview of each case or control was conducted by trained investigators to collect detailed individual information on sociodemographic characteristic (age, weeks of gestation, marital status, educational level, household income, etc.), lifestyle habits (dietary habits, smoking status, alcohol-drinking status, physical activity, etc.), menstrual history (number of pregnancies, fertility status, etc.), assessment of current pregnancy, family history, and medical history. Anthropometric measurements (weight, height, blood pressure) were measured before delivery by experienced medical personnel.

### Assessment of dietary fatty acid intake

We assessed the dietary intake of all participants with a 79-item semi-quantitative food frequency questionnaire (FFQ) of their diet during the three months prior to the study period^45^. Each food item had four options for frequency: 0 = never; 1 = per month; 2 = per week; 3 = per day. Each consumed component was assigned a corresponding frequency. The subjects spent 30–40 min recalling their food intake information. We provided the participants with photographs of food portion sizes to help them estimate the quantity of food intake. The amount of each food type consumed by the participant was calculated by multiplying the frequency with the corresponding component. The consumed nutrients (g/d or mg/d) and energy (kcal/d) were calculated based on the Chinese Food Composition Tables 2004, which include the nutrient portion and energy of each food item^46^. Some information was excluded, such as individuals whose energy intake was < 600 kcal or > 4000 kcal^47^ and those who ate < 4 food items, as their FFQs were considered incomplete^48^.

For dietary fatty acid intake, we calculated the intake of total fatty acids, SFAs, MUFAs, and PUFAs. We focused on n-3 PUFAs (ALA, DHA, EPA) and n-6 PUFAs (linoleic acid and AA).

### Statistical analysis

Relevant information about demographic characteristics, nutrients, and preeclampsia diagnosis were extracted from the participants’ responses to the FFQ. We described demographic and lifestyle characteristics and selected preeclampsia risk factors for the pregnant cases and controls. A paired *t-*test was used for continuous variables and McNemar’s test was used for categorical variables. All nutrients were adjusted for total energy intake using the residual method^49^. The differences between cases and controls were compared using a paired Wilcoxon signed-rank test.

The dietary intake of fatty acids was categorized into quartiles (Q1–Q4), and Q1 was defined as the reference group. The association between dietary fatty acid intake and the risk of developing preeclampsia was estimated using conditional logistic regression analysis in univariate and multivariate models. The potential confounders adjusted for in multivariate models included age (continuous, years), education level (primary school or less, secondary/high school, college/university or above), household income (≤ RMB2000, RMB2000–4000, RMB4000–6000, > RMB6000), pre-pregnancy body mass index (< 24 kg/m^2^, 24–27.9 kg/m^2^, ≥ 28 kg/m^2^), alcohol drinking (yes or no), passive smoking (yes or no), use of a multivitamin (yes or no), use of folic acid supplement (yes or no), daily energy intake (continuous, kcal/d), carbohydrate intake (continuous, kcal/d), and total cholesterol intake (continuous, kcal/d) using the enter method. Furthermore, linear trend tests were performed by converting the categorical indicator of fatty acids into continuous variables in univariate and multivariate models.

All data analyses were conducted using the Statistical Package for Social Sciences (SPSS) v20.0 (IBM Corporation, Armonk, NY, USA) and statistical significance was defined as a two-sided *p* value < 0.05. The sample size was calculated using Power Analysis and Sample Size (PASS) v11.0 (NCSS Corporation, UT, USA).

## Supplementary Information

Supplementary Information.

## Data Availability

The data used in this analysis are available from the corresponding author upon reasonable request.
