# Supplementary material for: The association between dietary fatty acid intake and the risk of developing preeclampsia: a matched case–control study
Source: Sci Rep. 2021 Feb 18;11:4048. doi: 10.1038/s41598-021-83674-3 (PMC7893000; doi:10.1038/s41598-021-83674-3)
Supplement: Supplementary file 1 — Supplementary Information. [file 41598_2021_83674_MOESM1_ESM.docx]

**Table S1** Risk of preeclampsia during pregnancy according to quartiles of dietary fatty acids intake by excluding participants with gestational diabetes mellitus.

|  | Quartiles of dietary energy-adjusted intake | | | | |
| --- | --- | --- | --- | --- | --- |
|  | Q1 | Q2 | Q3 | Q4 | *P*-trend |
| Total fatty acid |  |  |  |  |  |
| n (case/control) | 94/97 | 101/90 | 95/96 | 92/99 |  |
| Median, g/d (case/control) | 43.92/45.06 | 56.83/56.00 | 63.67/64.12 | 73.43/74.45 |  |
| Crude OR (95% CI) | 1.00 | 1.15 (0.78 - 1.71) | 1.03 (0.70 - 1.52) | 0.96 (0.65 - 1.43) | 0.708 |
| Adjusted OR (95% CI) | 1.00 | 1.34 (0.78 - 2.32) | 1.31 (0.71 - 2.43) | 1.16 (0.55 - 2.44) | 0.851 |
| SFA |  |  |  |  |  |
| n (case/control) | 100/91 | 99/92 | 93/98 | 90/101 |  |
| Median, g/d (case/control) | 12.33/12.40 | 15.49/15.52 | 17.50/17.73 | 20.59/20.74 |  |
| Crude OR (95% CI) | 1.00 | 0.97 (0.65 - 1.46) | 0.87 (0.58 - 1.31) | 0.83 (0.57 - 1.22) | 0.272 |
| Adjusted OR (95% CI) | 1.00 | 1.20 (0.71 - 2.01) | 1.21 (0.69 - 2.12) | 1.54 (0.83 - 2.87) | 0.197 |
| MUFA |  |  |  |  |  |
| n (case/control) | 97/94 | 87/104 | 101/90 | 97/94 |  |
| Median, g/d (case/control) | 16.24/17.38 | 22.49/22.78 | 26.44/26.68 | 32.45/32.36 |  |
| Crude OR (95% CI) | 1.00 | 0.81 (0.55 - 1.21) | 1.12 (0.74 - 1.70) | 1.00 (0.68 - 1.49) | 0.653 |
| Adjusted OR (95% CI) | 1.00 | 0.93 (0.55 - 1.60) | 1.24 (0.68 - 2.26) | 1.31 (0.67 - 2.56) | 0.259 |
| PUFA |  |  |  |  |  |
| n (case/control) | 100/91 | 83/108 | 100/91 | 99/92 |  |
| Median, g/d (case/control) | 14.50/14.34 | 18.76/18.59 | 22.69/22.81 | 29.58/28.42 |  |
| Crude OR (95% CI) | 1.00 | 0.70 (0.47 - 1.05) | 0.98 (0.66 - 1.47) | 1.01 (0.67 - 1.52) | 0.646 |
| Adjusted OR (95% CI) | 1.00 | 0.45 (0.27 - 0.75) | 0.79 (0.47 - 1.33) | 0.72 (0.41 - 1.29) | 0.756 |
| Ratio of MUFA to SFA |  |  |  |  |  |
| n (case/control) | 98/93 | 86/105 | 89/102 | 109/82 |  |
| Median (case/control) | 1.15/1.15 | 1.40/1.39 | 1.57/1.57 | 1.87/1.85 |  |
| Crude OR (95% CI) | 1.00 | 0.78 (0.53 - 1.16) | 0.85 (0.56 - 1.28) | 1.27 (0.84 - 1.93) | 0.224 |
| Adjusted OR (95% CI) | 1.00 | 0.63 (0.39 - 1.01) | 0.63 (0.38 - 1.03) | 1.00 (0.60 - 1.66) | 0.906 |

Abbreviation: OR, odds ratio; CI, confidence interval; SFA, saturated fatty acids; MUFA, mono-unsaturated fatty acids; PUFA, polyunsaturated fatty acids.

Crude and adjusted OR (95% CI): from conditional logistic model. Covariates includes age, gestational weeks, education level, household income, pre-pregnancy body mass index, alcohol drinking, passive smoking, use of multivitamin, use of folic acid supplement, daily energy intake, carbohydrate intake and total cholesterol intake by enter method.

**Table S2** Risk of preeclampsia during pregnancy according to quartiles of different kinds of polyunsaturated fatty acids by excluding participants with gestational diabetes mellitus.

|  | Quartiles of dietary energy-adjusted intake | | | | |
| --- | --- | --- | --- | --- | --- |
|  | Q1 | Q2 | Q3 | Q4 | *P*-trend |
| n-6 PUFAs |  |  |  |  |  |
| n (case/control) | 94/97 | 93/98 | 94/97 | 101/90 |  |
| Median, g/d (case/control) | 9.66/9.89 | 13.31/13.40 | 16.17/16.68 | 21.18/21.70 |  |
| Crude OR (95% CI) | 1.00 | 0.97 (0.65 - 1.45) | 0.99 (0.66 - 1.47) | 1.16 (0.78 - 1.73) | 0.485 |
| Adjusted OR (95% CI) | 1.00 | 1.04 (0.63 - 1.70) | 0.81 (0.49 - 1.34) | 1.10 (0.64 - 1.89) | 0.974 |
| LA |  |  |  |  |  |
| n (case/control) | 94/97 | 93/98 | 94/97 | 101/90 |  |
| Median, g/d (case/control) | 9.61/9.76 | 13.20/13.33 | 16.10/16.55 | 21.07/21.60 |  |
| Crude OR (95% CI) | 1.00 | 0.97 (0.65 - 1.45) | 0.99 (0.66 - 1.47) | 1.16 (0.78 - 1.73) | 0.486 |
| Adjusted OR (95% CI) | 1.00 | 1.02 (0.62 - 1.67) | 0.80 (0.48 - 1.32) | 1.11 (0.65 - 1.90) | 0.985 |
| AA |  |  |  |  |  |
| n (case/control) | 114/77 | 99/92 | 88/103 | 81/110 |  |
| Median, mg/d (case/control) | 25.01/22.72 | 55.29/57.28 | 76.75/75.80 | 111.03/114.10 |  |
| Crude OR (95% CI) | 1.00 | 0.74 (0.49 - 1.11) | 0.60 (0.40 - 0.89) | 0.52 (0.35 - 0.78) | 0.001 |
| Adjusted OR (95% CI) | 1.00 | 0.89 (0.51 - 1.56) | 0.75 (0.38 - 1.49) | 0.85 (0.30 - 2.45) | 0.529 |
| n-3 PUFAs |  |  |  |  |  |
| n (case/control) | 99/92 | 94/97 | 89/102 | 100/91 |  |
| Median, g/d (case/control) | 0.52/0.58 | 1.21/1.24 | 1.94/1.96 | 2.93/2.87 |  |
| Crude OR (95% CI) | 1.00 | 0.92 (0.62 - 1.36) | 0.82 (0.56 - 1.22) | 1.03 (0.68 - 1.54) | 0.948 |
| Adjusted OR (95% CI) | 1.00 | 1.05 (0.66 - 1.67) | 0.78 (0.49 - 1.24) | 0.98 (0.59 - 1.62) | 0.616 |
| ALA |  |  |  |  |  |
| n (case/control) | 99/92 | 95/96 | 88/103 | 100/91 |  |
| Median, g/d (case/control) | 0.52/0.58 | 1.22/1.22 | 1.93/1.95 | 2.93/2.86 |  |
| Crude OR (95% CI) | 1.00 | 0.94 (0.63 - 1.40) | 0.81 (0.54 - 1.20) | 1.03 (0.68 - 1.55) | 0.895 |
| Adjusted OR (95% CI) | 1.00 | 1.08 (0.67 - 1.73) | 0.75 (0.47 - 1.19) | 0.97 (0.59 - 1.60) | 0.527 |
| EPA |  |  |  |  |  |
| n (case/control) | 118/73 | 103/88 | 93/98 | 68/123 |  |
| Median, mg/d (case/control) | 0.27/0.30 | 2.05/1.92 | 4.91/5.34 | 13.75/13.18 |  |
| Crude OR (95% CI) | 1.00 | 0.72 (0.47 - 1.10) | 0.58 (0.38 - 0.88) | 0.33 (0.22 - 0.52) | <0.001 |
| Adjusted OR (95% CI) | 1.00` | 0.77 (0.47 - 1.26) | 0.60 (0.37 - 0.97) | 0.36 (0.21 - 0.60) | <0.001 |
| DHA |  |  |  |  |  |
| n (case/control) | 114/77 | 97/94 | 90/101 | 81/110 |  |
| Median, mg/d (case/control) | 0.16/0.11 | 1.77/1.96 | 4.41/4.18 | 13.12/13.13 |  |
| Crude OR (95% CI) | 1.00 | 0.67 (0.44 - 1.02) | 0.59 (0.39 - 0.90) | 0.49 (0.32 - 0.74) | 0.001 |
| Adjusted OR (95% CI) | 1.00 | 0.73 (0.44 - 1.20) | 0.72 (0.43 - 1.20) | 0.57 (0.35 - 0.93) | 0.034 |
| n-3/n-6 PUFAs |  |  |  |  |  |
| n (case/control) | 98/93 | 97/94 | 85/106 | 102/89 |  |
| Median (case/control) | 0.03/0.03 | 0.09/0.08 | 0.13/0.13 | 0.23/0.24 |  |
| Crude OR (95% CI) | 1.00 | 0.99 (0.66 - 1.50) | 0.75 (0.50 - 1.13) | 1.12 (0.73 - 1.70) | 1.000 |
| Adjusted OR (95% CI) | 1.00 | 1.10 (0.66 - 1.83) | 0.69 (0.42 - 1.12) | 1.22 (0.75 - 2.00) | 0.907 |

Abbreviation: PUFAs, polyunsaturated fatty acids; OR, odds ratio; CI, confidence interval; LA, linoleic acid; AA, arachidonic acid; ALA, alpha-linolenic acid; EPA, eicosapentaenoic acid; DHA, docosahexaenoic acid.

Crude and adjusted OR (95% CI): from conditional logistic model. Covariates includes age, gestational weeks, education level, household income, pre-pregnancy body mass index, alcohol drinking, passive smoking, use of multivitamin, use of folic acid supplement, daily energy intake, carbohydrate intake and total cholesterol intake by enter method.
